# Gambling-related attitudes and dimensional structure of the GABS-15 in outpatient addiction care patients: associations with gambling disorder

**DOI:** 10.3389/fpsyt.2024.1481733

**Published:** 2024-10-21

**Authors:** Andreas M. Bickl, Johanna K. Loy, Ludwig Kraus, Bettina Grüne, Eva Hoch, Larissa Schwarzkopf

**Affiliations:** ^1^ IFT Institut für Therapieforschung, Centre for Mental Health and Addiction Research, Munich, Germany; ^2^ Department of Child and Adolescent Psychiatry, Psychosomatics, and Psychotherapy, University of Cologne, Faculty of Medicine and University Hospital Cologne, Cologne, Germany; ^3^ Department of Public Health Sciences, Centre for Social Research on Alcohol and Drugs, Stockholm University, Stockholm, Sweden; ^4^ Centre of Interdisciplinary Addiction Research (ZIS), Department of Psychiatry and Psychotherapy, University Medical Centre Hamburg-Eppendorf, Hamburg, Germany; ^5^ Institute of Psychology, ELTE, Eötvös Loránd University, Budapest, Hungary; ^6^ German Youth Institute (Deutsches Jugendinstitut e.V. (DJI)), Munich, Germany; ^7^ Department of Psychiatry and Psychotherapy, University Hospital of Munich, Munich, Germany; ^8^ Charlotte Fresenius University, Munich, Germany; ^9^ Pettenkofer School of Public Health, Munich, Germany

**Keywords:** addictive behavior, addiction, gambling, pathological gambling, gambling related beliefs and attitudes, longitudinal

## Abstract

**Introduction:**

Individuals with gambling disorder (GD) harbor cognitive distortions and dysfunctional beliefs about gambling that may foster problematic gambling behaviour. Evidence on particularly detrimental attitudes and beliefs is however lacking. To close this knowledge gap, we analysed associations between gambling attitudes and beliefs (Gambling Attitudes and Beliefs Survey (GABS-15)) and severity of gambling disorder (DSM-5 criteria met) in a German cohort of 123 individuals receiving outpatient gambling treatment.

**Methods:**

Data from the "Katamnese-Study" covering a 36-months timeframe with 5 assessment points was pooled. The multidimensional structure of the GABS-15 was examined using explorative and confirmatory factor analyses, followed by mixed-effect regression models using different operationalizations of the GABS-15.

**Results:**

A three-factorial structure comprising “attitudes while gambling”, “sensation-seeking / excitement”, and "gambling fallacies” demonstrated better fit indices than the GABS-15 sum score. Only the "gambling fallacies " factor (1.00, p<0.05; 15.36, p < 0.01) was significantly associated with increased severity of GD.

**Discussion:**

As a one-dimensional conceptualization of gambling-related attitudes and beliefs might not sufficiently guide staff of outpatient treatment facilities regarding priority setting in gambling care, evidence on attitudes with particularly detrimental associations is highly warranted. Here, focussing on mitigating "gambling' fallacies" by tailored treatment strategies appears promising.

## Introduction

Gambling disorder (GD), a behavioral addiction characterized by uncontrollable gambling (1) causes societal harm as well as substantial psychological, social, and financial strain (2). Individuals with GD experience lower quality of life, work, and health, and often face high rates of psychiatric comorbidity compared to non-gamblers (2, 3).

Gambling behavior is triggered by gambling-related attitudes (4, 5), emotions [e. g., arousal, emotional excitement, see (6, 7)], as well as cognitive biases and irrational beliefs (4, 8, 9), which are crucial factors in the development and maintenance of problem gambling and GD (9, 10). Cognitive biases and irrational beliefs stem from an erroneous attribution of random game outcomes to a presumed but not existing cause-and-effect relationship. For instance, players may attribute favorable outcomes to personal skills and unfavorable ones to bad luck. The concept of “gambler’s fallacy”, meaning that players expect deviations from chance (such as prolonged losses) to eventually balance out and correct themselves in subsequent sequences, relies on similar assumptions (11, 12).

These misconceptions also support the development of specific beliefs, gambling strategies, and behaviors aiming at exerting control over game outcomes (13). Gamblers often adopt specific attitudes that they assume to influence the game’s outcome and to increase the chance of winning. These include being cautious and composed regardless of the situation (whether winning or losing), exuding confidence during the game, and, for some, even gambling with passion (4, 14, 15). Additionally, in some cases, superstitious beliefs come into play. These are understood as a strong conviction that arises from the mistaken perception of a cause-and-effect relationship between two unrelated events, leading to the belief that certain rituals or the presence of “lucky charms” can influence the outcome of a game and contribute to success (16).

Individuals with gambling problems are more likely to endorse irrational beliefs about gambling (17) and to experience increased illusions of control (18, 19). Furthermore, findings indicate a correlation between positively valued attitudes to gambling and GD, perpetuating a misguided sense of control (20, 21).

A widely used tool to assess cognitive distortions in gamblers is the Gambling Attitudes and Beliefs Survey (GABS) (5), which assesses “gambling affinity” via a one-dimensional sum score created from 35 items on cognitive biases, irrational beliefs, and positively valued attitudes to gambling. GABS and its 15-items short version (GABS 15) (22), have been demonstrated to be associated with gambling behavior (23), to discriminate between problem and non-problem gamblers, to capture behavioral and cognitive changes during inpatient treatment (24), and to predict relapse (25, 26) as well as recovery (27).

However, concerns have been raised about the one-dimensionality claim. Factor analyses revealed a multidimensional structure for both the original GABS (5 factors: strategies, chasing, attitudes, luck, and emotions) (15) and the GABS-15 (3 factors: sensation seeking/illusion of control, luck/gambler’s fallacy, and attitude/emotions) (28). Our study aims to 1) broaden the pre-existing body of evidence on the GABS-15’s factorial structure and 2) investigate how the factorial structure interacts with problem gambling.

## Methods

### Design and setting

Data were collected as part of the “Katamnese-Study”, a prospective, naturalistic cohort study conducted in 28 Bavarian outpatient addiction care facilities (OACF) between 2014 and 2019. Participants completed a composite diagnostic interview at admission and written questionnaires at admission and at 6-, 12-, 24-, and 36-month follow-ups. These data were linked to individual routine documentation from the German Addiction Care Statistical Service. Further details on study design, instruments used, and methodology have been published elsewhere (29).

### Study sample

Adults receiving treatment for GD, possessing proficient German language skills, and engaging in a minimum of three interactions with their corresponding OACF were eligible for inclusion in the study. Recruitment took place between December 2014 and August 2016. 145 clients provided informed consent and participated in the baseline assessment. From this initial pool, 22 individuals were excluded from the final analysis due to missing baseline information on the GABS-15 (n = 6), gambling-related problems (n = 2) and/or treatment termination at follow-up 3 (n = 7). Additionally, participants still undergoing treatment at follow-up 3 (n = 7) were excluded, resulting in a final sample size of 123 participants. Detailed information on the study’s sampling procedure has been published elsewhere (30, 31).

#### Measures of interest

*Gambling-related problems* during the 12 months prior to baseline and the period between the distinct follow-up assessments were assessed using a German translation of the DSM-IV-oriented “Stinchfield criteria” (hereafter: GD criteria questionnaire), a validated tool to measure the severity of GD (32). The more recent DSM-5 classification categorizes GD as mild (4 – 5 criteria met), moderate (6 – 7 criteria met), and severe (8 – 9 criteria met). To align the GD criteria questionnaire with DSM-5 guidelines, those items about the eighth criterion concerning illegal activities were excluded. Thus, the adapted GD criteria questionnaire assesses eight of the nine DSM-5 criteria for GD by two dummy-coded items and the remaining ninth criterion (“withdrawal”) by one dummy-coded item. Each DSM-5 criterion was considered fulfilled if at least one of the associated items was affirmed. The total number of endorsed DSM-5 criteria was summed up at each assessment point (baseline, follow-up 1, follow-up 2, follow-up 3, follow-up 4), resulting in a GD score ranging from 0 to 9 which reflects severity of GD according to DSM-5.

*Gambling-related attitudes and beliefs* were measured by the German version of the GABS-15 (33) (**Supplementary Table 1**). The GABS-15 is a 15 item, forced-choice instrument capturing cognitive biases, irrational beliefs, degree of subjective arousal, and excitement experienced through gambling, as well as positively valued attitudes towards gambling. Each item is measured on a 4-point Likert scale (1 = “strongly disagree” to 4 = “strongly agree”). Item-specific scores are summed up resulting in a total score of 15 to 60 points, with higher scores indicating more substantial cognitive distortions.

#### Covariables

*Comorbid mental disorders* were assessed at baseline via the Composite International Diagnostic Interview (CIDI) (34). We considered affective disorders (yes/no), an umbrella term for major depression, dysthymic disorder, and bipolar disorders (35), and anxiety disorders (yes/no), an umbrella term for panic disorder, agoraphobia, specific phobia, social phobia, generalized anxiety disorder, unspecified anxiety disorder, obsessive-compulsive disorder, and post-traumatic stress disorder (35).

*Electronic gambling machines (EGMs)* represent one of the detrimental forms of gambling and were reported to be extensively used by individuals seeking help (36, 37). Thus, participation in EGM plays was incorporated as a dichotomized covariable (yes/no).

M*igration background* has been identified as a risk factor for the less favorable development of GD (30, 38). Thus, self-reported migration background (yes/no) according to the definition provided by the Federal Statistical Office of the Federal Republic of Germany was accounted for. This means that a migration background exists if individuals had migrated to Germany themselves or if one of their (grand)parents had immigrated prior to their birth (39).

Furthermore, we collected self-reported information if *GD-related help* had already been sought prior to the current help-seeking episode (yes/no). Self-reported age (in years) and sex (male/female) were used as standard demographic covariates.

### Ethical statement

All ethical protocols, including those related to data protection and participant well-being, were strictly adhered to in the conduct of this research. The research project received ethical clearance from the German Association for Psychology (Deutsche Gesellschaft für Psychologie - DGPs) under the reference LK 092014 on November 3, 2014, indicating its compliance with ethical standards.

### Statistical analysis

Using our longitudinal data, we pooled all individual observations over the distinct assessment points and conducted exploratory factor analyses (EFA) on the GABS-15 items using varimax rotation. Kaiser-Meyer-Olkin index, Bartlett test of sphericity, Kaiser-Guttmann criterion, and scree plot inspection were used for interpreting the factor structure. Additionally, Cronbach’s Alpha was computed to evaluate internal consistency. Factor scores were saved for further analyses.

To evaluate the compatibility of our empirically derived factor solution with the one-dimensional factor structure suggested by Breen and Zuckerman (5) and the three-factor structure proposed by Gehlenborg and colleagues (28), we conducted confirmatory factor analyses (CFA) using the Chi-square test, comparative fit index (CFI), Tucker–Lewis index (TLI), root mean square error of approximation (RMSEA), and standardized root mean square residual (SRMR) for comparison and interpretation.

To investigate the association between the identified dimensions and severity of GD, we applied three mixed-effects regression models that compared a) the GABS-15 sum score [model 1; Breen and Zuckermann (5)], b) the three-factor solution suggested by Gehlenborg et al. (28) (model 2), and c) our empirically derived solution (model 3).

To address individual variation in changes over time and to account for intra-subject correlation in the context of repeated measurements, all models incorporated time and participant ID as random effects. Furthermore, all analyses were adjusted for the presence of affective (reference: no) or anxiety disorders (reference: no), migration background (reference: no), EGM-play at baseline (reference: no), GD-related help sought prior to the current help-seeking episode (reference: no), age, and sex (reference: male). Considering the small sample size, we performed 1,000 non-parametric bootstrap replications to obtain more accurate parameter estimates and measures of uncertainty [i. e., 95%-confidence intervals (CI) (40)].

To assess the robustness of our findings, we repeated our models in a sensitivity analysis using gambling behavior as an alternative outcome. Here gambling behavior was represented as weekly gambling hours. This parameter was derived as a multiplicative index using information on gambling frequency (average number of gambling days per week) and gambling intensity (average number of hours spent per gambling day) in the previous 12 months (for baseline) and the time since the last assessment point for each follow-up.

All statistical analyses were conducted using Stata/SE 15 (Stata Corp LP; College Station, TX, USA).

## Results

### Study participation and participants’ baseline characteristics

Of the 123 individuals included, 73.2% (*n* = 90) participated at follow-up 1, 62.6% (*n* = 77) at follow-up 2, 55.3% (*n* = 68) at follow-up 3, and 49.6% (*n* = 61) at follow-up 4. 43.9% (*n* = 54) responded at all four follow-ups.

As summarized in **Table 1**, at baseline, study participants were on average 35.5 (SD = 10.8) years old, 86.2% (n = 106) were male, and 30.9% (n = 38) had a migration background. 55.1% (n = 59) were diagnosed with an affective disorder and 33.6% (n = 36) with an anxiety disorder. 88.4% (n = 107) had previously sought GD-related help and 76.0% (n = 92) reported gambling on EGMs. The average GABS-15 score was 35.7 (SD = 8.0). Agreement to the distinct GABS-15 Items is visualized in **Supplementary Figure 1**.

**Table 1:** Participant demographics, comorbid mental disorders, GABS-15, and gambling characteristics.

Variables	Baseline (*n* = 123)	
**Age in years years,** *M*, (*SD*)	35.5	(10.8)
**Male Gender,** *n*, (*%*)	106	(86.2%)
**Migration background,** *n*, (%)	38	(30.9%)
**GD-related help sought before the study,** *n*, (%)	107	(88.4%)
**Playing on EGMs,** *n* (%)	92	(76.0%)
Comorbid mental disorders		
**GABS-15^1^,** *M, (SD)*	35.7	(8.0)
*Affective disorders, n*, (%)	59	(55.1%)
*Anxiety disorders, n*, (%)	36	(33.6%)
**Severity of GD**		
*GD score* ***^2^*** *M*, (*SD*)	7.9	(1.2)
Severity grades		
GD Criteria < 4 *n, (%)*	1	(0.8%)
Mild (4-5 criteria) *n, (%)*	3	(2.4%)
Moderate (6-7 criteria) *n, (%)*	39	(31.7%)
Severe (8-9 criteria) *n, (%)*	80	(65.0%)
Gambling behaviour (n=114)		
*Gambling frequency (gambling days per week) M, (SD)*	3.7	(1.7)
*Gambling intensity (spent hours gambling per gambling day) M, (SD)*	6.9	(3.5)
*Gambling behavior index M, (SD)*	27.0	(20.8)

GABS-15, Gambling Attitudes and Beliefs Survey-15; GD, gambling disorder; EGM, electronic gambling machines.

Due to space constraints, the opposite categories for variables with binary (yes/no) options are not displayed as they can be inferred from the reverse probabilities.

1GABS-15 score: unweighted sum of all GABS-15 items; 2GD score (measure of gambling-related problems): sum of no. of fulfilled DSM-5 criteria for GD based on participant endorsement of criteria (via yes-no question).

The average GD score was 7.9 (SD = 1.2), with 2.4% (n = 3) of participants meeting the criteria for mild GD, 31.7% (n = 39) for moderate GD, and 65.0% (n = 80) for severe GD. The participants gambled on average 3.7 days (SD = 1.7) per week and 6.9 hours (SD = 3.5) per gambling day corresponding to 27.0 weekly gambling hours (SD = 20.8).

### Factor analysis

**Supplementary Table 2** presents the item-total correlations among GABS items and Cronbach’s alpha coefficients of the GABS-15 scale. The criteria for conducting an EFA were met, as indicated by a Kaiser-Meyer-Olkin index of 0.94. Additionally, a significant Bartlett test (χ2(105) = 3440.54, p < 0.001) supported the assumption of sphericity. Applying the Kaiser-Guttmann criterion (eigenvalue > 1), three factors were extracted, collectively explaining 92.2% of the total variance. We labelled the first dimension “gambling fallacies” (explaining 40.0% of the variance after rotation), the second dimension “attitudes while gambling” (explaining 30.1% of the variance after rotation), and the third dimension “sensation seeking/excitement” (explaining 22.1% of the variance after rotation). **Table 2** provides the factor loadings for all 15 items.

**Table 2:** Loadings of GABS-15 items on the three dimensions from the varimax-rotated factor analysis.

Items	Gambling fallacies	Attitudes while Gambling	Sensation/Excitement
**2.** If I have not won any of my bets for a while, I am probably due for a big win	**0.704**	0.330	
**3.** I know when I am on a streak	**0.558**	0.411	
**15.** If I have lost my bets recently, my luck is bound to change	**0.705**		0.307
**4.** When I gamble, it is important to act as if I am calm even if I am not		**0.672**	
**5.** It is important to feel confident when I gamble		**0.648**	
**6.** People who gamble are more daring and adventurous than those who never gamble			**0.590**
**8.** If you have never experienced the excitement of making a big bet, you have never really lived	0.396		**0.641**
**1.** Gambling makes me feel really alive	0.453	0.331	0.320
**7.** Sometimes I just know I am going to have good luck	0.485	0.330	0.422
**9.** No matter what the game is, there are betting strategies that can help you win	0.429		0.311
**10.** If I lose at gambling, it is important to stay calm		0.494	
**11.** If I have been lucky lately, I should press my bets	0.476	0.488	
**12.** I must be familiar with a gambling game if I am going to win		0.334	
**13.** Some people can bring bad luck to other people	0.373		
**14.** To be successful at gambling, I must be able to identify streaks	0.441	0.367	0.328

Loadings > .5 are set in bold type. Only factor loadings > .3 are presented. Items are ordered based on factor loadings. Item numbering refers to the original item order of the GABS-15.

Three items loaded on the first dimension (all loadings > 0.5), two items on the second dimension (all loadings > 0.6), and two items on the third dimension (all loadings > 0.5).

Within the CFA, our three-factor model (χ2(11) = 43.53, p < 0.001, CFI = 0.98, TLI = 0.96, RMSEA = 0.08, SRMR = 0.03) demonstrated superior fit compared to both the conventional GABS-15 sum score (χ2(90) = 455.88, p < 0.001, CFI = 0.89, TLI = 0.87, RMSEA = 0.10, SRMR = 0.05) and the three-factor model proposed by Gehlenborg and colleagues (χ2(74) = 280.01, p < 0.001, CFI = 0.93, TLI = 0.92, RMSEA = 0.08, SRMR = 0.04).

### Comparison of the longitudinal associations of GABS-15 and GD

As depicted in **Table 3**, we found a significant association between the GABS-15 sum score (model 1) and severity of GD (0.14, p < 0.01). For Gehlenborg’s three-factor structure (model 2), we observed a significant association only for the ‘luck/gambler’s fallacy’ factor (1.94, p < 0.01). Also, in our empirically derived three-factor model (model 3), only the ‘gambling fallacies’ factor (1.00, p < 0.05) presented a statistically significant association.

**Table 3:** Comparison of mixed-effects regression estimates for associations between GABS-15 score, factor scores based on Gehlenborg et al. (28) and factor scores based on pooled data with covariables on GD-score.

Variables	Severity of GD		
	Model 1 (Breen & Zuckermann)	Model 2 (Gehlenborg et al.)	Model 3 (Pooled data)
**GABS-15 score^1^**	0.14*** (95%-CI: 0.10 – 0.19)		
Gehlenborg et al. (28)			
Sensation seeking/illusion of control		-0.45 (95%-CI: -1.96 – 1.06)	
Luck/gambler’s fallacy		1.94** (95%-CI: 0.13 – 3.75)	
Attitude/emotions		-0.12 (95%-CI: -0.91 – 0.67)	
Pooled data solution			
Gambling fallacies			1.00** (95%-CI: 1.00 – 1.89)
Attitudes while gambling			0.08 (95%-CI: -0.58 – 0.74)
Sensation/excitement			0.25 (95%-CI: -0.46 – 0.96)
Covariables			
Age	0.00 (95%-CI: -0.03 – 0.03)	0.01 (95%-CI: -0.03 – 0.04)	0.00 (95%-CI: -0.03 – 0.04)
Gender (male)	0.03 (95%-CI: -0.95 – 1.00)	0.14 (95%-CI: -0.90 – 1.17)	0.00 (95%-CI: -1.10 – 1.10)
Migration background (yes)	0.60* (95%-CI: -0.02 – 1.23)	0.59* (95%-CI: -0.04 – 1.21)	0.62* (95%-CI: -0.02 – 1.27)
GD-related help sought before the study (yes)	-0.76 (95%-CI: -1.93 – 0.41)	-0.95 (95%-CI: -2.15 – 0.25)	-0.74 (95%-CI: -1.91 – 0.43)
Playing on EGMs	1.31*** (95%-CI: 0.53 – 2.08)	1.34*** (95%-CI: 0.54 – 2.13)	1.42*** (95%-CI: 0.62 – 2.21)
Comorbid mental disorders			
Affective disorders	0.17 (95%-CI: -0.59 – 0.94)	0.18 (95%-CI: -0.56 – 0.92)	0.12 (95%-CI: –0.67 – 0.91)
Anxiety disorders	- 0.05 (95%-CI: -0.75 – 0.64)	0.10 (95%-CI: -0.63 – 0.83)	0.10 (95%-CI: –0.62 – 0.82)

GABS-15, Gambling Attitudes and Beliefs Survey-15; GD, gambling disorder; EGM, electronic gambling machine. CI, Confidence interval.

**^1^** GABS-15 score: unweighted sum of all GABS-15 items; **^2^** GD score (measure of gambling-related problems): sum of no. of fulfilled DSM-5 criteria for GD based on participant endorsement of criteria (via yes-no question).

*p<0.1, **p<0.05, ***p<0.01

#### Sensitivity analysis

The sensitivity analysis included 114 participants who provided information on gambling frequency and intensity. Generally, the GABS-15 sum score (model 1) and the factors derived from model 2 and model 3 were more pronouncedly associated with weekly gambling hours than with severity of GD (**Supplementary Table 3**). We observed notable increases in effect sizes regarding GABS-15 sum score (model 1; 0.76, p < 0.01), the ‘luck/gambler’s fallacy’ factor (2; 24.75, p < 0.01) in model 2, and the ‘gambling fallacies’ factor (15.36, p < 0.01) in model 3. Additionally, both the sensation seeking/illusion of control factor (-11.54, p < 0.10) and the attitude/emotions factor (-6.72, p < 0.10) of model 2 displayed negative associations with weekly gambling hours.

## Discussion

Using longitudinal data collected over a 3-year timeframe, the primary objective of this paper was to analyze the dimensional framework of the GABS-15 within an outpatient cohort of individuals with GD. Additionally, we sought to investigate to which extent personal attitudes and beliefs contribute to individual differences course GD over time. Contravening the previously stated one-dimensional structure of GABS-15, our analysis suggests a three-dimensional structure comprising “gambling fallacies”, “attitudes while gambling”, and “sensation-seeking/excitement”. Focusing on presumed associations of gambling-related attitudes and beliefs with the severity of GD, our empirically derived three-factor structure outperformed the (one-factorial) GABS-15 sum score, with “gambling fallacies” being the most indicative factor.

Considering that the GABS and the GABS-15 are less frequently used in gambling research than other measurement tools and that they had been mainly applied in samples largely consisting of undergraduates (41), evidence on “archetypic” sum scores across diverse populations is sparse. The baseline GABS-15 sum score of our sample closely mirrors the sum scores observed among individuals engaging in self-guided interventions or metacognitive training for gambling problems (e. g., 42, 43). Hence, our sample apparently resembles other populations of help-seeking individuals with gambling-related problems in an outpatient setting.

Like us, a previous study examining the dimensional structure of the GABS-15 revealed three distinct factors, namely “sensation seeking/illusion of control”, “luck/gambler’s fallacy”, and “attitude/emotions” (28). These factors apparently capture similar aspects as those derived from our sample, albeit with slightly different factor compositions. Despite differences in the factor composition both three-factorial concepts (Gehlenborg structure, our empirically derived structure) presented slightly better fit indices than the (one-factorial) GABS-15 sum score. This supports the rising body of evidence questioning the initially claimed one-dimensional structure of the GABS and the GABS 15 (15, 23, 28).

Out of the three identified factors only the factor “gambling fallacies” demonstrated a significant association with the severity of GD. This was also the case when the factors of the Gehlenborg model were included in the regression analysis. Consistent with these results, a meta-analytical investigation of the connection between problem gambling and various cognitive bias assessment instruments revealed that subscales explicitly addressing ‘gambler’s fallacy’ consistently exhibited stronger and more robust associations with problem gambling than other biases (41). One potential rationale for this observation could be that the predominant use of EGMs was popular in our sample. EGMs have been demonstrated to foster the development of gambling fallacies and excitement (44, 45), as they present past gambling events in an immediate and sequential manner. This format supports elements (i.e., sequential observation and recency effects) that contribute to the emergence of gambling fallacies (46). Hence, the role of the ‘gambling fallacies’ factor might be less prominent in samples of non-EGM players.

The large share of EGM players might also explain that only items unrelated to gambling strategies or illusions of control exerted a significant impact on the factors linked to cognitive distortions or excitement within our sample. Evidence suggests that among EGM players, illusions of control are less influential compared to superstitious beliefs and the elicitation of positive emotions (16). Building on Trivedi & Reichert’s (47) study of online gamblers, which identified a negative correlation between the use of gambling strategies and the severity of gambling problems, our negative findings may reflect a similar pattern in our predominantly EGM player sample. It is likely that these players are primarily focused on smaller, immediate rewards, and that more individual gambling strategies may be perceived negatively when subjected to personal reflection. Moreover, strategic aspects in gambling seem to have a lesser role, particularly when compared to skill-based forms of gambling such as poker or sports-betting (28, 48).

Within our analyses, the associations with gambling fallacies became even more accentuated when gambling behavior instead of the severity of GD was looked at. Contrary to our expectations our sensitivity analysis also revealed mitigating associations for the factors “sensation seeking/illusion of control” and “attitude/emotions” of the Gehlenborg structure. Although we do not have a definitive explanation for this counterintuitive result, it is possible that delay discounting—referring to the tendency to devalue future, larger rewards in favor of smaller, immediate ones—played a role in the observed gambling behavior (47).

### Limitations and strengths

When interpreting the results of our study the following caveats must be kept in mind: First, even though we exceeded the suggested minimum sample size of n >=50 for modelling psychometric properties of socio-psychological constructs (49), the small sample size employed for the EFA stage of our analysis may have favored an inaccurate factor structure and factor solution. Through pooling individual-level data from distinct assessment points, we increased the number of observations which supposedly mitigated issues of a small sample size. However, further validation of the factors derived based on larger and more diverse samples is needed to enhance generalizability and reliability of the findings. Second, the comparative fit indices of the three models contrasted within our CFA must be interpreted with caution. As we identified and validated our factors within the same data set it is not surprising that our model structure outperformed Gehlenborg’s structure and the GABS-15 sum score. Nevertheless, it is quite common to perform CFA and EFA within the same sample (28, 50, 51). Furthermore, our prior interest was to investigate associations of factors with indicators for problem gambling (severity of GD, weekly gambling hours) and not to best possibly explain pre-existing gambling-related attitudes and beliefs within the observed sample. Hence, we consider the chosen approach – which is not unimpeachable from a methodological point of view – justified. Third, the observational one-armed design of our study does not allow drawing strictly causal relationships. Indeed, a bidirectional relationship between gambling fallacies and GD was suggested: Thus, gambling fallacies can exacerbate problematic gambling behavior, but conversely, they can also develop and strengthen as sequelae of intensified gambling (8, 52).

The strength of our study on the other hand lies in our comprehensive approach that not only explored the connections of gambling-related attitudes and beliefs with problem severity but also delved into actual gambling behavior. This was achieved by employing multilevel mixed models on two outcomes, enabling an assessment based on an intersubjective perspective. Moreover, the naturalistic approach in our study facilitated a degree of generalizability among individuals seeking outpatient care for GD. This contrasts with other studies that exclusively focused on isolated clinical samples and supports external validity by encompassing a diverse group of individuals. Finally, by contrasting our factor model with two alternative models, we indirectly performed a kind of cross-validation of previous work, improving our knowledge of the factor structure of the GABS-15.

## Conclusions and further research

While the creators of the GABS emphasize its one-dimensional nature, intended to measure a general affinity for gambling, our findings – in alliance with previous studies – suggest that the GABS reveals greater potential when approached from a multidimensional perspective. This enables researchers, counsellors, and therapists to focus on distinct cognitive distortions that are strongly correlated with severity and course of GD which in turn might support effective treatment. In this regard, adequate addressing of gambling fallacies appears to be of high therapeutic relevance.

## Data Availability

The raw data supporting the conclusions of this article will be made available by the authors, without undue reservation.
