# Supplementary material for: Gambling-related attitudes and dimensional structure of the GABS-15 in outpatient addiction care patients: associations with gambling disorder
Source: Front Psychiatry. 2024 Oct 21;15:1481733. doi: 10.3389/fpsyt.2024.1481733 (PMC11532057; doi:10.3389/fpsyt.2024.1481733)
Supplement: Supplementary file 1 [file Table1.docx]

| **Table A1:** Original English language items in the GABS-15 |
| --- |
| **Original 15 items in the GABS-15 (in English)** |
| *1. Gambling makes me feel really alive.* |
| *2. If I have not won any of my bets for a while, I am probably due for a big win.* |
| *3. I know when I am on a streak.* |
| 4. When I gamble, it is important to act as if I am calm even if I am not. |
| 5. It is important to feel confident when I gamble. |
| *6. People who gamble are more daring and adventurous than those who never gamble.* |
| *7. Sometimes I just know I am going to have good luck.* |
| *8. If you have never experienced the excitement of making a big bet, you have never really lived.* |
| *9. No matter what the game is, there are betting strategies that can help you win.* |
| *10. If I lose at gambling, it is important to stay calm.* |
| *11. If I have been lucky lately, I should press my bets.* |
| *12. I must be familiar with a gambling game if I am going to win.* |
| *13. Some people can bring bad luck to other people.* |
| *14. To be successful at gambling, I must be able to identify streaks.* |
| *15. If I have lost my bets recently, my luck is bound to change.* |

**Figure B1:** Agreement to the distinct GABS-15 items

**Table A2:** Item-total correlations and Cronbach's alpha coefficients of the GABS-15 scale

| GABS-15 | Item-total correlations | Alpha if item is deleted | Alpha of scale |
| --- | --- | --- | --- |
| GABS-15 Items |  |  | **0.87** |
| 1. Gambling makes me feel really alive | 0.59 | 0.86 |  |
| 2. If I have not won any of my bets for a while, I am probably due for a big win | 0.72 | 0.86 |  |
| 3. I know when I am on a streak | 0.68 | 0.86 |  |
| 4. When I gamble, it is important to act as if I am calm even if I am not | 0.51 | 0.87 |  |
| 5. It is important to feel confident when I gamble | 0.56 | 0.86 |  |
| 6. People who gamble are more daring and adventurous than those who never gamble | 0.59 | 0.86 |  |
| 7. Sometimes I just know I am going to have good luck | 0.66 | 0.86 |  |
| 8. If you have never experienced the excitement of making a big bet, you have never really lived | 0.65 | 0.86 |  |
| 9. No matter what the game is, there are betting strategies that can help you win | 0.55 | 0.87 |  |
| 10. If I lose at gambling, it is important to stay calm | 0.28 | 0.88 |  |
| 11. If I have been lucky lately, I should press my bets | 0.60 | 0.86 |  |
| 12. I must be familiar with a gambling game if I am going to win | 0.51 | 0.87 |  |
| 13. Some people can bring bad luck to other people | 0.58 | 0.86 |  |
| 14. To be successful at gambling, I must be able to identify streaks | 0.74 | 0.85 |  |
| 15. If I have lost my bets recently, my luck is bound to change | 0.73 | 0.86 |  |

| **Table A3:** Sensitivity analysis: Comparison of mixed-effects regression estimates for associations between GABS-15 score, factor scores based on Gehlenborg et al. 2022 and factor scores based on pooled data with covariables on weekly gambling hours | | | |
| --- | --- | --- | --- |
|  | **Severity of GD** | | |
| **Variables** | **Model 1 (Breen & Zuckermann)** | **Model 2 (Gehlenborg et al.)** | **Model 3 (Pooled data)** |
| **GABS-15 score^1^** | 0.76*** (95%-CI: 0.45 – 1.06) |  |  |
| **Gehlenborg et al. 2022** |  |  |  |
| Sensation seeking/illusion of control |  | -11.54* (95%-CI: -24.43 – 1.34) |  |
| Luck/gambler’s fallacy |  | 24.75*** (95%-CI: 10.08 – 39.42) |  |
| Attitude/emotions |  | -6.72* (95%-CI: -14.27 – 0.83) |  |
| **Pooled data solution** |  |  |  |
| Gambling fallacies |  |  | 15.36*** (95%-CI: 7.14 – 23.58) |
| Attitudes while gambling |  |  | -4.85 (95%-CI: -11.01 – -1.31) |
| Sensation / excitement |  |  | -3.77 (95%-CI: -8.85 – 1.32) |
| **Covariables** |  |  |  |
| Age | -0.24* (95%-CI: -0.48 – 0.01) | -0.18 (95%-CI: -0.42 – 0.06) | -0.16 (95%-CI: -0.40 – 0.08) |
| Gender (male) | -3.18 (95%-CI: -14.4 – 8.09) | -0.37 (95%-CI: -10.77 – 10.03) | -0.23 (95%-CI: -11.04 – 10.57) |
| Migration background (yes) | -2.84 (95%-CI: -8.73 – 3.06) | -4.28 (95%-CI: -10.33 – 1.78) | -4.36 (95%-CI: -10.22 – 1.50) |
| GD-related help sought before the study (yes) | -12.68 (95%-CI: -29.94 - 4.55) | -15.47* (95%-CI: -32.00 – 1.05) | -13.11 (95%-CI: -28.81 – 2.59) |
| Playing in EGMs | -3.50 (95%-CI: -944 – 2.45) | -3.52 (95%-CI: -9.38 – 2.35) | -3.50 (95%_CI: -9.31 – 2.31) |
| Comorbid mental disorders |  |  |  |
| Affective disorders | 1.98 (95%-CI: -3.07 – 7.03)) | 2.84 (95%-CI: -1.90 – 7.57) | 2.26 (95%-CI: -2.48 – 7.00) |
| Anxiety disorders | -4.83 (95%-CI: -10.67 – 1.01) | -2.78 (95%-CI: -9.33 – 3.77) | -3.03 (95%-CI: -8.94 – 2.89) |
| *Note.* GABS-15 = Gambling Attitudes and Beliefs Survey-15; GD = gambling disorder; EGM = electronic gambling machine. CI = Confidence interval  **^1^** GABS-15 score: unweighted sum of all GABS-15 items; **^2^**GD score (measure of gambling-related problems): sum of no. of fulfilled DSM-5 criteria for GD based on participant endorsement of criteria (via yes-no question).  *p<0.1, **p<0.05, ***p<0.01 | | | |
